# Supplementary material for: Activation of EGFR signaling by Tc-Vein and Tc-Spitz regulates the metamorphic transition in the red flour beetle Tribolium castaneum
Source: Sci Rep. 2021 Sep 22;11:18807. doi: 10.1038/s41598-021-98334-9 (PMC8458297; doi:10.1038/s41598-021-98334-9)
Supplement: Supplementary file 1 — Supplementary Figures. [file 41598_2021_98334_MOESM1_ESM.docx]

**Supplementary Figures**

**Activation of EGFR signaling by Tc-Vein and Tc-Spitz regulates the metamorphic transition in the red flour beetle *Tribolium castaneum***

Sílvia Chafino, David Martín* and Xavier Franch-Marro*

Institute of Evolutionary Biology (IBE, CSIC-Universitat Pompeu Fabra), Passeig de la Barceloneta 37, 08003 Barcelona, Catalonia, Spain.

* Authors for correspondence: [xavier.franch@ibe.upf-csic.es](mailto:xavier.franch@ibe.upf-csic.es) and david.martin@ibe.upf-csic.es

**Supplementary Figure 1.** Amino acid sequence of *Tribolium* Tc-Vn. The signal peptide is underlined. The Ig-like domain is shown in bold blue and the Egfr-like domain is represented in bold green. The PEST region is marked in bold black between conserved protein domains. The highly-conserved cysteines are represented in bold red.

**
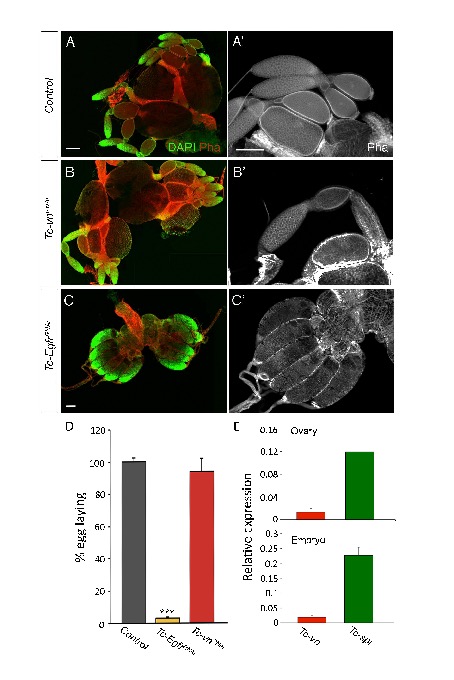
Supplementary Figure 2. Roles of Tc-Egfr and Tc-Vn in oogenesis of *Tribolium.*** Comparison of ovaries from females treated with (A-A’) *dsMock* (control)*,* (B-B’) *dsTc-Egfr* (*dsTc-Egfr^RNAi^*) and (C-C’) *dsTc-vn* (*dsTc-vn^RNAi^*). The ovaries are stained with Phalloidin (in red) and DAPI (in green) to visualize the structure of the tissue and the nuclei of the cells respectively. Scale bar represents 150 μm. (G) Percentage of egg laying in *Tc-Egfr^RNAi^* and *Tc-vn^RNAi^* female adults compared with *Control*. Error bars indicate the SEM (n=3). (E) *Tc-vn* and *Tc-spi* mRNA levels measured by qRT-PCR in ovary and embryo. Transcript abundance values are normalized against the *Tc-Rpl32* transcript.
