# Supplementary material for: Activation of EGFR signaling by Tc-Vein and Tc-Spitz regulates the metamorphic transition in the red flour beetle Tribolium castaneum
Source: Sci Rep. 2021 Sep 22;11:18807. doi: 10.1038/s41598-021-98334-9 (PMC8458297; doi:10.1038/s41598-021-98334-9)
Supplement: Supplementary file 2 — Supplementary Tables. [file 41598_2021_98334_MOESM2_ESM.docx]

**Supplementary Tables**

**Activation of EGFR signaling by Tc-Vein and Tc-Spitz regulates the metamorphic transition in the red flour beetle *Tribolium castaneum***

Sílvia Chafino, David Martín* and Xavier Franch-Marro*

Institute of Evolutionary Biology (IBE, CSIC-Universitat Pompeu Fabra), Passeig de la Barceloneta 37, 08003 Barcelona, Catalonia, Spain.

* Authors for correspondence: [xavier.franch@ibe.upf-csic.es](mailto:xavier.franch@ibe.upf-csic.es) and david.martin@ibe.upf-csic.es

**S1 Table.** Phenotypes of *T. castaneum* injected with *dsTc-Egfr^RNAi^, dsTc-pnt^RNAi^* and *dsTcEgfr^RNAi^*+*20E* in the last larval instar.

| **Treatment^a^** | **n** | **L7**  **arrested** | **Prepupal**  **arrest** | **Pupa** | **Pupal**  **arrest** |
| --- | --- | --- | --- | --- | --- |
| *Control* | 50 | - | - | 50 (100%) | - |
| *dsTc-Egfr^RNAi^* | 60 | - | 60 (100%) | - | **-** |
| *dsTc-pnt^RNAi^* | 30 | - | 24 (80%) | - | 6 (20%) |
| *dsTcEgfr^RNAi^*+*20E* | 16 | - | 10 (62,5%) | 6 (37,5%) | **-** |

^a^ The *dsRNAs* were injected in penultimate instar larvae (L6), and the phenotypes were scored on the larval-pupal transition.

**S2 Table.** Phenotypes of *T. castaneum* injected with *dsTc-Egfr^RNAi^* in penultimate and last larval instars.

| **Treatment^a^** | **n** | **L6**  **arrested** | **L7**  **arrested** | **Prepupal**  **arrest** | **Pupa** |
| --- | --- | --- | --- | --- | --- |
| *Control* | 30 | - | - | - | 30 (100%) |
| *dsTc-Egfr^RNAi^* | 30 | - | - | 30 (100%) |  |

^a^ The *dsRNAs* were injected in antepenultimate instar larvae (L5), and the phenotypes were scored on the larval-pupal transition.

**S3 Table.** Phenotypes of *T. castaneum* injected with *dsTc-spi^RNAi^* in the last larval instar.

| **Treatment^a^** | **n** | **L7**  **arrested** | **Prepupal arrest** | **Pupa** | **Pupal**  **arrest** |
| --- | --- | --- | --- | --- | --- |
| *Control* | 40 | - | - | 40 (100%) | - |
| *dsTc-spi^RNAi^* | 38 | - | **-** | 38 (100%) | **-** |

^a^ The *dsRNAs* were injected in penultimate instar larvae (L6), and the phenotypes were scored on the larval-pupal transition.

**S4 Table.** Accession numbers of Vein sequences used for alignments.

**S5 Table.** Phenotypes of *T. castaneum* injected with *dsTc-vn^RNAi^* and *dsTc-vn^RNAi^+dsTc-spi^RNAi^* in the last larval instar.

| **Treatment^a^** | **n** | **L7**  **arrested** | **Prepupal**  **arrest** | **Pupa** | **Pupal**  **arrest** |
| --- | --- | --- | --- | --- | --- |
| *Control* | 37 | - | - | 37 (100%) | - |
| *dsTc-vn^RNAi^* | 26 | - | 3 (11,6%) | 23 (88,4%) | **-** |
| *dsTc-vn^RNAi^+*  *dsTc-spi^RNAi^* | 24 | - | 24 (100%) | - | **-** |

^a^ The *dsRNAs* were injected in penultimate instar larvae (L6), and the phenotypes were scored on the larval-pupal transition.
